# Can 3D radiological calculations predict operational difficulties for rectal cancer?: A single center retrospective analysis

**DOI:** 10.1097/MD.0000000000036961

**Published:** 2024-01-19

**Authors:** Necdet Fatih Yasar, Elif Gundogdu, Arda Sakir Yilmaz, Bartu Badak, Fatma Didem Bayav, Alaattin Ozen, Setenay Oner

**Affiliations:** a Department of General Surgery, Faculty of Medicine, Osmangazi University, Eskişehir, Turkey; b Department of Radiology, Faculty of Medicine, Osmangazi University, Eskişehir, Turkey; c Department of General Surgery, Yunus Emre State Hospital, Eskişehir, Turkey; d Department of General Surgery, Faculty of Medicine, Osmangazi University, Eskişehir, Turkey; e Department of Radiation Oncology, Faculty of Medicine, Osmangazi University, Eskişehir, Turkey; f Department of Biostatistics, Faculty of Medicine, Eskisehir Osmangazi University, Eskişehir, Turkey.

**Keywords:** low anterior resection, pelvimetry, rectal cancer, tumor volume

## Abstract

Low anterior resection, performing total mesorectal excision with appropriate pelvic dissection to prevent local recurrence, is probably the most challenging type of surgery in colorectal surgery, especially in a narrow pelvis. In this study, we aimed to predict the operation difficulty of rectal cancer by comparing the operation time with 2D and 3D pelvimetry. Sixty-six patients who underwent total mesorectal excision after neoadjuvant chemoradiotherapy due to primary rectal cancer located in the middle and lower rectum (10 cm from the anus) were included in the study. Surgery notes were reviewed and data on demographic factors, tumor stage, duration of surgery, and types of surgery were collected, as well as pelvimetric parameters. All protocols had 2D T2-weighted sequences in 3 planes (axial, sagittal, and coronal). Pelvimetric measurements were made by measuring 8 pelvic lengths and 2 angles. Pelvis and tumor volume were measured by manual margin monitoring. In each slice, both pelvis and tumor boundaries were manually drawn individually in the sagittal plane. Pelvis and tumor volumes were calculated from the set of adjacent images by summing slice thickness and products of area measurements within the pelvis and tumor boundaries. In our results, no correlation was observed with operation time, including pelvic volume. Exception for this were interacetabular distance and tumor volume. In the regression test, the only parameter that correlated with the operation time was tumor volume. In conclusion, we believe that tumor volumetric calculations may be useful in predicting difficult distal rectal carcinoma surgeries.

## 1. Introduction

Low anterior resection (LAR) is probably the most challenging type of operation in colorectal surgery, especially in the narrow pelvis. Proper pelvic dissection with total mesorectal excision (TME) is vital to prevent local recurrence.^[1]^ New strategies such as robotic surgery and transanal TME have been proposed to overcome this technical difficulty.^[2–4]^ However, these techniques cost more and require advanced surgical skills and technological infrastructure. Therefore, the importance of forming selection criteria for difficult cases emerges.

Although it is still under debate, pelvimetry has been proposed as a helpful tool in determining surgical difficulties in rectal cancer resection.^[5–7]^ However, the studies are controversial about the effectiveness of these radiologic predictors.^[2,8]^ Many of these studies on pelvimetry include only 2-dimensional calculations. There are only a few studies with 3-dimensional analyses, which do not include pelvic volume but only tumor volume.^[9]^

In this study, we investigated the correlations between the operative time, as an indicator of the difficulty of rectal cancer surgery, and 3- and 2-dimensional pelvimetry and demographic parameters.

## 2. Material and methods

Ethical approval for this retrospective study was obtained from the local research ethics committee (Eskişehir Osmangazi University Ethics Committee IRB: 2020-11-32). In total, the medical reports of 82 patients with primary rectal cancer, who were treated between January 2019 and December 2021, were retrospectively analyzed. Sixty-six patients who underwent TME following neoadjuvant chemoradiotherapy due to primary rectal cancer located in the middle and lower rectum (10 cm from the anal verge) were included. The operations were performed by 2 senior surgeons experienced in colorectal surgery. Patient position was optimized in each case. Patients who had cancer located in the upper rectum or rectosigmoid junction tumor, who underwent partial mesorectal excision without receiving prior neoadjuvant therapy, who had recurrent rectal cancer, and who had additional procedures such as metastasectomy, cholecystectomy, etc, and previous abdominal procedures were excluded. Additionally, patients who did not have preoperative pelvic magnetic resonance imaging (MRI) were excluded as well. The operation notes were reviewed and data on demographic factors, tumor stage, operation time, and types of operation were collected besides the pelvimetric parameters. The patients were analyzed according to the operation types including, LAR (open LAR), lap LAR (laparoscopic LAR), APR (open abdomino-perineal resection), lap APR (laparoscopic APR), and conversion (conversion to open surgery) groups. In all LAR cases including laparoscopy and open technique, the double-stapling technique was preferred while all the APRs were performed in the lithotomy position.

### 2.1. Neoadjuvant chemoradiotherapy (NCRT)

The patients with the T2-T4 low rectal cancer, T4 middle rectal cancers, and T3 middle rectal cancer with node positivity or extramural vascular invasion were included. All patients have completed a long-course NCRT with a total radiation dose of 45 to 50.4 Gy delivered in daily fractions of 1.8 to 2 Gy over a 5- to 6-week period combined with 5-fluorouracil or capecitabine.

### 2.2. MRI pelvimetry

All MRI examinations were performed using 3 Tesla (General Electric, Milwaukee, WI) or 1.5 Tesla (Siemens, Berlin, Germany) MRI devices with body coils 6 to 8 weeks after completion of NCRT and used for tumor restaging and tumor response evaluation. All protocols had 2D T2 weighted sequences in 3 planes (axial, sagittal, and coronal). The axial and coronal T2-weighted sequences were angled perpendicular to the tumor axis as defined on sagittal T2-weighted images. The slice thickness for all planes was 3 mm without an intersection gap.

The MR images were evaluated by the 2 radiologists who were blinded to the patients’ clinical and histopathological information with a consensus. Using a dedicated workstation (GE, Advantage Workstation 4.7, USA) all measurements were made twice, and average values were used for analysis. Sagittal and axial T2-weighted sequences were used for all pelvimetric measurements (length, angle, and length of anterior sacrococcygeal curve), tumor, and pelvic volume. All measurements were made using free hand-drawn irregular “regions of interest” for every section.

**Figure 1.**
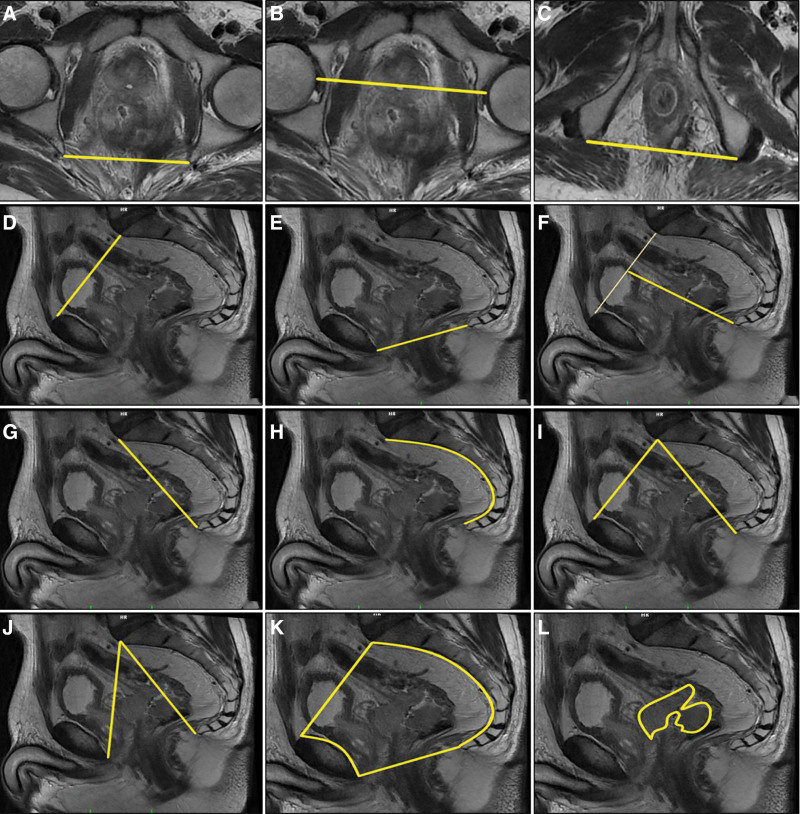
Schematic representation of the main pelvimetry measurement, tumor, and pelvis volume on the MRI. (A) Interichiatic spinous distance. (B) Interacetabular distance. (C) Intertuberous distance. (D) Promontory to pubic symphysis distance (pelvic inlet length). (E) Pubic symphysis to the tip of the coccyx distance (pelvic outlet length). (F) Mid-inlet length (pelvic depth). (G) Promontory to coccyx distance. (H) Length of anterior sacrococcygeal curve. (I) Promontory to the top of the pubic symphysis angle. (J) Promontory to the lowest tip of the public symphysis angle. (K) Pelvic volume. (L) Tumor volume. MRI = magnetic resonance imaging.

Pelvimetric measures included 8 pelvic lengths A: the interischial distance [distance between the ischial spines], B: the interacetabular distance [distance between the innermost points of femoral heads], C: the intertuberous distance [distance between the innermost points of the ischial tuberosities], D: the pelvic inlet length [the distance between the upper border of the pubic symphysis and the promontory], E: the pelvic outlet length [the distance between the lower border of the pubic symphysis and the coccyx], F: the depth of the pelvis [distance from the midpoint of the pelvic inlet measured distance (promontory to pubic symphysis distance) to the lower end of the coccyx], G: the distance between the promontory and the coccyx, H: the length of sacrococcygeal curve, and 2 angles (I-Promontory to the top of the pubic symphysis angle, J-Promontory to the lowest tip of the public symphysis angle), were measured as shown in Figure 1.

Pelvis and tumor volume were done by manual boundary tracing method. Both pelvis and tumor boundaries were manually drawn on the sagittal plane one by one on each slice. Pelvis and tumor volumes were calculated from the set of contiguous images by summing the products of the area measurements within the pelvis and tumor boundaries and slice thickness. Volumes were obtained automatically with volume-specific software (Advantage Work Station AW 4.7 software, GE Healthcare, WI). We also calculated the ratio between the pelvic volume and the depth of pelvis.

### 2.3. Statistics

The pelvimetric and the volumetric parameters and body mass indexes were found to be normally distributed using the Shapiro–Wilk test and the independent samples *t* test was used in further analysis. To compare the effects of gender and tumor stage on the operation duration, *t* test and 1-way ANOVA test were used, respectively. Statistical significance was denoted by *P* < .05. The relationships between pelvic and volumetric parameters, and operative time were studied with Pearson correlations followed by regression analysis of significant variables.

## 3. Results

We used operative time as an objective, validated indicator of operative difficulty,^[2]^ and studied the relations between the parameters and operation time.

Mean and standard deviation values of age, length of hospital stay, body mass index, and all measured parameters are shown in Table 1.

**Table 1:** Age, gender, length of hospital stay, body mass index, and mean and standard deviation values of all measured parameters.

	Mean (n = 66)	Std. deviation
Age	64.292	9.297
Male/female	44/22	(2:1)
Body mass index (kg/m^2^)	27.557	3.379
Operation time (dk)	276.853	63.718
Length of stay in hospital (day)	9.107	2.610
Pelvic volume (cm^3^)	823.625	112.574
Tumor volume (cm^3^)	32.426	19.887
Interischial distance (cm)	9.969	0.775
Interacetabular distance (cm)	11.782	0.565
Intertuberous distance (cm)	12.269	0.816
Pelvic inlet length (cm)	11.627	0.851
Pelvic outlet length (cm)	8.810	0.698
Depth of pelvis (cm)	11.216	0.909
Distance between the promontory and the coccyx (cm)	12.590	1.195
The length of sacrococcygeal curve (cm)	15.828	1.215
Promontory to the top of the pubic symphysis angle (^o^)	63.822	5.273
Promontory to the lowest tip of the public symphysis angle (^o^)	39.663	3.969
Pelvic volume/ depth of pelvis	2.867	1.692

Gender and tumor stage did not affect the operative time. Body mass index (BMI) had a correlation with operative time only in the LAR group (Table 2).

**Table 2:** Correlation between operation time with gender, BMI, and tumor stage.

	General (n = 66)	LAR (n = 28)	Lap. LAR (n = 14)	APR (n = 14)	Lap. APR (n = 6)	Conversion (n = 4)
Gender	*t* = 1.566 *P* = .122	*t* = 1.046 *P* = .305	*t* = −0.287 *P* = .781	*t* = 0.887 *P* = .388	*t* = 1.225 *P* = .266	*t* = 0.139 *P* = .902
Body mass index	*R* = 0.161 *P* = .183	***R* = 0.296** ***P* = .013**	*R* = 0.001 *P* = .998	*R* = 0.008 *P* = .976	*R* = 0.417 *P* = .304	*R* = −0.327 *P* = .673
Tumor stage	*F* = 0.094 *P* = .911	*F* = 0.200 *P* = .820	*F* = 0.946 *P* = .433	*F* = 0.756 *P* = .487	*F* = 0.529 *P* = .619	*F* = 0.472 *P* = .717

APR = abdomino-perineal resection, LAR = low anterior resection.

The sole pelvic parameter that correlated with the operative time was interacetabular distance (*R* = 0.264, *P* = .027). Among the pelvimetric and volumetric parameters, only the tumor volume was correlated with the operative time (*R* = 0.433, *P* < .001). Besides the tumor volume, pelvic volume correlated with the operative time better than the other parameters, but the correlation did not reach statistical significance (*R* = 0.088, *P* = .205) (Table 3). Subgroup analysis of the patients according to the type of operation showed that only tumor volume correlated with the operative time. The Lap APR group was the only exception to this, in which pelvic volume had a better correlation with the operative time (*R* = 0.705, *P* = .031). The relation between the ratio of pelvic volume to pelvic depth and operation time did not perform any additional significant correlation than pelvic volume (Table 4).

**Table 3:** Correlation between operation time with pelvimetric measurements and tumor volume.

	General (n = 66)	LAR (n = 28)	Lap. LAR (n = 14)	APR (n = 14)	Lap. APR (n = 6)	Conversion (n = 4)
Interischial distance	*R* = 0.089 *P* = .486	*R* = 0.168 *P* = .634	*R* = 0.348 *P* = .493	*R* = 0.711 *P* = .192	*R* = 0.372 *P* = .327	*R* = 0.167 *P* = .612
Interacetabular distance	***R* = 0.264** ***P* = .027**	*R* = 0.357 *P* = .053	*R* = 0.219 *P* = .543	*R* = −0.341 *P* = .166	*R* = 0.143 *P* = .736	*R* = 0.136 *P* = .864
Intertuberous distance	*R* = −0.023 *P* = .850	*R* = 0.071 *P* = .711	*R* = −0.169 *P* = .641	*R* = −0.146 *P* = .564	*R* = −0.305 *P* = .463	*R* = 0.763 *P* = .237
Pelvic inlet length	*R* = 0.077 *P* = .529	*R* = −0.156 *P* = .411	*R* = −0.266 *P* = .458	*R* = 0.409 *P* = .092	*R* = 0.392 *P* = .337	*R* = 0.066 *P* = .934
Pelvic outlet length	*R* = −0.008 *P* = .944	*R* = −0.330 *P* = .075	*R* = 0.331 *P* = .351	*R* = 0.141 *P* = .578	*R* = 0.398 *P* = .329	*R* = −0.579 *P* = .421
Depth of pelvis	*R* = 0.055 *P* = .649	*R* = −0.174 *P* = .357	*R* = 0.328 *P* = .355	*R* = 0.110 *P* = .663	*R* = 0.625 *P* = .097	*R* = −0.435 *P* = .565
Distance between the promontory and the coccyx	*R* = 0.088 *P* = .471	*R* = −0.121 *P* = .523	*R* = 0.103 *P* = .777	*R* = 0.145 *P* = .567	*R* = 0.551 *P* = .157	*R* = −0.020 *P* = .980
The length of sacrococcygeal curve	*R* = 0.162 *P* = .179	*R* = 0.091 *P* = .633	*R* = 0.042 *P* = .909	*R* = 0.189 *P* = .452	*R* = 0.511 *P* = .196	*R* = −0.313 *P* = .687
Promontory to the top of the pubic symphysis angle	*R* = −0.100 *P* = .409	*R* = 0.068 *P* = .720	*R* = 0.250 *P* = .486	*R* = −0.264 *P* = .290	*R* = −0.367 *P* = .371	*R* = −0.750 *P* = .250
Promontory to the lowest tip of the public symphysis angle	*R* = −0.170 *P* = .159	*R* = −0.206 *P* = .274	*R* = −0.213 *P* = .554	*R* = −0.199 *P* = .429	*R* = 0.037 *P* = .930	***R* = −0.975** ***P* = .025**
Pelvic volume	*R* = 0.088 *P* = .205	*R* = −0.084 *P* = .659	*R* = 0.067 *P* = .854	*R* = 0.099 *P* = 0.402	***R* = 0.754** ***P* = .031**	*R* = −0.157 *P* = .843
Tumor volume	***R* = 0.433** ***P* < .001**	***R* = 0.381** ***P* = .038**	***R* = 0.901** **p=<0.001**	***R* = 0.475** ***P* = .046**	*R* = 0.685 *P* = .061	***R* = 0.983** ***P* = .017**
Pelvic volume/depth of pelvis	*R* = 0.191 *P* = .114	*R* = −0.011 *P* = .952	*R* = −0.070 *P* = .847	*R* = 0.401 *P* = .099	***R* = 0.771** ***P* = .025**	*R* = −0.046 *P* = .954

APR = abdomino-perineal resection, LAR = low anterior resection.

**Table 4:** Regression analysis of volumetric measurements.

	General	LAR	Lap. LAR	APR	Lap. APR
Interacetabular distance	*t* = 1.335 *P* = .803	*t* = 1.957 *P* = .061	*t* = 0.221 *P* = .832	*t* = 0.519 *P* = .612	*t* = −1.102 *P* = .332
Tumor volume	***t* = 3.526** ***P* = .001**	*t* = 1.901 *P* = .068	***t* = 5.072** ***P* = .002**	*t* = 1.172 *P* = .261	*t* = 0.602 *P* = .580
Pelvic volume	*t* = 0.440 *P* = .662	*t* = −1.292 *P* = .208	*t* = −0.559 *P* = .597	*t* = 0.336 *P* = .742	*t* = 2.166 *P* = .096

APR = abdomino-perineal resection, LAR = low anterior resection.

The conversion of the laparoscopic operation to open surgery might indicate operative difficulty. However, there was no difference between the laparoscopy and the conversion groups in terms of volumetric, pelvimetric, and demographic parameters.

## 4. Discussion

The studies are controversial about the effectiveness of these radiologic predictors in showing the difficulty of the operation.^[5–9]^ This study has indicated that volumetric calculations may be more helpful in deciding on the difficulty level of the rectal carcinoma case.

Recently, it has been proposed that the robotic approach may provide a better mesorectal specimen and local control compared to the laparoscopic technique in male patients with mid-low rectal cancer while the same research group has failed to show these benefits in female patients.^[10,11]^ This suggests the robotic surgery may be superior, especially in the narrow pelvis. However, robotic surgery is expensive and difficult to access; selection criteria for rectal carcinoma cases may increase the benefits and cost-effectiveness of this technique.

The previous literature is mainly focused on the relation between pelvimetric parameters with operative time and circumferential resection margin (CRM) positivity.^[5–8]^ While operative timing is an objective indicator of the difficulty of an operation, CRM positivity may indicate not only the difficulty but the quality as well. However, the number of patients in this study was not enough to make any claim on any relation between pelvimetry and CRM positivity. Therefore, we are focused on operative time.

One of the main biases in the previous studies was the selection criteria of the patients. Most of the previous studies disregarded the location of the rectal tumor, which obviously may have affected both operative times.^[5–7]^ Another bias of the prior studies was that there was no specific information on patients who received preoperative radiotherapy or did not.^[5–7]^ In our study, we included only the patients with distal rectal carcinoma who received neoadjuvant chemoradiotherapy to investigate a more homogenous population.

Many of the studies on pelvimetry included only 2-dimensional calculations and came up with very different findings.^[5–8]^ Killeen et al reported that a larger pelvic outlet and a larger angle between the promontory, S3, and the coccygeal tip were both related to longer operations. Furthermore, they examined the ratios of the measurements and found that a higher sagittal pelvic inlet:intertuberous distance ratio and a higher sagittal pelvic outlet:intertuberous distance ratio might have increased the operative time.^[6]^ On the other hand, Ferko et al^[3]^ suggested that the angle between the longitudinal axis of the symphysis, and the lines between the symphysis and the promontory might affect the CRM status. Instead of angles, Boyle et al^[5]^ proposed that the anteroposterior diameter of the inlet, the anteroposterior diameter of the midplane, and the transverse diameter of the midplane might be related to the CRM positivity. Controversially, some studies failed to demonstrate any correlation between pelvimetric parameters, and operative time, and CRM positivity.^[2,8]^ The only pelvic parameter associated with the operative time in our analysis was interacetabular distance but in the regression test, we did not observe any correlation between interacetabular distance and operative time.

Escal et al^[12]^ proposed an MRI-based scoring system with 4 items (BMI, type of surgery, intertuberous distance, and mesorectal fat area) to predict surgical difficulty in rectal cancer patients. The European MRI and Rectal Cancer Surgery study group reported a new scoring system (body mass index, inter-spinous distance, γmrT stage, and male sex) with higher accuracy.^[13]^ Of these 2 scoring systems, the only shared parameter was BMI, which correlated with operative time in the LAR group in our study. However, we failed to demonstrate any correlation with the other parameters of both scoring systems.

The European MRI and Rectal Cancer Surgery study group has also investigated the predictors of surgical outcomes and found that tumor volume was associated with operative time as we did.^[9]^ As expected, it could be harder to resect a tumor in a narrow pelvis with greater depth. However, we did not observe any relation between the depth and operation time. On the other hand, pelvic volume seemed to be correlated with the operative time better than the other pelvimetric parameters, even if it did not reach any significance. Furthermore, we failed to show a relation between the operation time and pelvic volume in the regression test. The only parameter that had a correlation with operation time in the regression test was tumor volume. It can be proposed that a deep and narrow pelvis may have the same volume as a wide but shallow pelvis, which might have interfered with our results. To overcome this bias, we also studied the relation between the ratio of pelvic volume to pelvic depth and operation time and again did not observe any correlation.

The first limitation of the present study was the retrospective design. We excluded the patients who underwent surgery with additional extra interventions and the patients with a history of previous abdominal surgery. However, it is impossible to exclude everyday life factors which might have affected our results. In addition, no robotic TME cases were included in our study, so no comparison could be made. The relatively small sample size was another limitation of the study. Multicentric, prospective observational studies with larger sample sizes may verify our results.

As a result, we believe that volumetric calculations of the tumor can be useful to predict difficult distal rectal carcinoma surgeries.

## Author contributions

**Conceptualization:** Necdet Fatih Yasar, Arda Sakir Yilmaz.

**Data curation:** Arda Sakir Yilmaz.

**Formal analysis:** Necdet Fatih Yasar, Bartu Badak, Setenay Oner.

**Investigation:** Arda Sakir Yilmaz, Bartu Badak, Alaattin Ozen.

**Methodology:** Necdet Fatih Yasar, Elif Gundogdu, Arda Sakir Yilmaz, Alaattin Ozen.

**Project administration:** Necdet Fatih Yasar.

**Software:** Arda Sakir Yilmaz, Fatma Didem Bayav, Setenay Oner.

**Supervision:** Bartu Badak.

**Validation:** Arda Sakir Yilmaz, Bartu Badak, Alaattin Ozen.

**Visualization:** Elif Gundogdu, Fatma Didem Bayav.

**Writing – review & editing:** Necdet Fatih Yasar, Elif Gundogdu, Setenay Oner.

## References

[1] Heald RJ Ryall RD. Recurrence and survival after total mesorectal excision for rectal cancer. Lancet. 1986;1:1479–82. 2425199 10.1016/s0140-6736(86)91510-2

[2] Baek SJ Kim CH Cho MS. Robotic surgery for rectal cancer can overcome difficulties associated with pelvic anatomy. Surg Endosc. 2015;29:1419–24. 25159651 10.1007/s00464-014-3818-x

[3] Ferko A Malý O Örhalmi J. CT/MRI pelvimetry as a useful tool when selecting patients with rectal cancer for transanal total mesorectal excision. Surg Endosc. 2016;30:1164–71. 26123334 10.1007/s00464-015-4324-5

[4] Butterworth JW Butterworth WA Meyer J. A systematic review and meta-analysis of robotic-assisted transabdominal total mesorectal excision and transanal total mesorectal excision: which approach offers optimal short-term outcomes for mid-to-low rectal adenocarcinoma? Tech Coloproctol. 2021;25:1183–98. 34562160 10.1007/s10151-021-02515-7

[5] Boyle KM Petty D Chalmers AG. MRI assessment of the bony pelvis may help predict resectability of rectal cancer. Colorectal Dis. 2005;7:232–40. 15859960 10.1111/j.1463-1318.2005.00819.x

[6] Killeen T Banerjee S Vijay V. Magnetic resonance (MR) pelvimetry as a predictor of difficulty in laparoscopic operations for rectal cancer. Surg Endosc. 2010;24:2974–9. 20464426 10.1007/s00464-010-1075-1

[7] Salerno G Daniels IR Brown G. Magnetic resonance imaging pelvimetry in 186 patients with rectal cancer confirms an overlap in pelvic size between males and females. Colorectal Dis. 2006;8:772–6. 17032323 10.1111/j.1463-1318.2006.01090.x

[8] Salerno G Daniels IR Brown G. Variations in pelvic dimensions do not predict the risk of circumferential resection margin (CRM) involvement in rectal cancer. World J Surg. 2007;31:1313–20. 17468974 10.1007/s00268-007-9007-5

[9] de’Angelis N Pigneur F Martínez-Pérez A. Predictors of surgical outcomes and survival in rectal cancer patients undergoing laparoscopic total mesorectal excision after neoadjuvant chemoradiation therapy: the interest of pelvimetry and restaging magnetic resonance imaging studies. Oncotarget. 2018;9:25315–31. 29861874 10.18632/oncotarget.25431 PMC5982752

[10] Aliyev V Goksel S Bakir B. Sphincter-saving robotic total mesorectal excision provides better mesorectal specimen and good oncological local control compared with laparoscopic total mesorectal excision in male patients with mid-low rectal cancer. Surg Technol Int. 2021;38:160–6. 33537982

[11] Aliyev V Piozzi GN Huseynov E. Robotic male and laparoscopic female sphincter-preserving total mesorectal excision of mid-low rectal cancer share similar specimen quality, complication rates and long-term oncological outcomes. J Robot Surg. 2023;17:1637–44. 36943657 10.1007/s11701-023-01558-2

[12] Escal L Nougaret S Guiu B. MRI-based score to predict surgical difficulty in patients with rectal cancer. Br J Surg. 2018;105:140–6. 29088504 10.1002/bjs.10642

[13] de’Angelis N Pigneur F Martínez-Pérez A. Assessing surgical difficulty in locally advanced mid-low rectal cancer: the accuracy of two MRI-based predictive scores. Colorectal Dis. 2019;21:277–86. 30428156 10.1111/codi.14473

